# Supplementary material for: Transcriptome Analysis Reveals Vimentin-Induced Disruption of Cell–Cell Associations Augments Breast Cancer Cell Migration
Source: Cells. 2022 Dec 13;11(24):4035. doi: 10.3390/cells11244035 (PMC9776984; doi:10.3390/cells11244035)
Supplement: Supplementary file 1 [file cells-11-04035-s001.zip › DEGs and enrichment analysis.zip!/DEGs and enrichment analysis/WT_VIMvsVector KEGG/ALL/WT_VIMvsVector_all.bar.pdf]

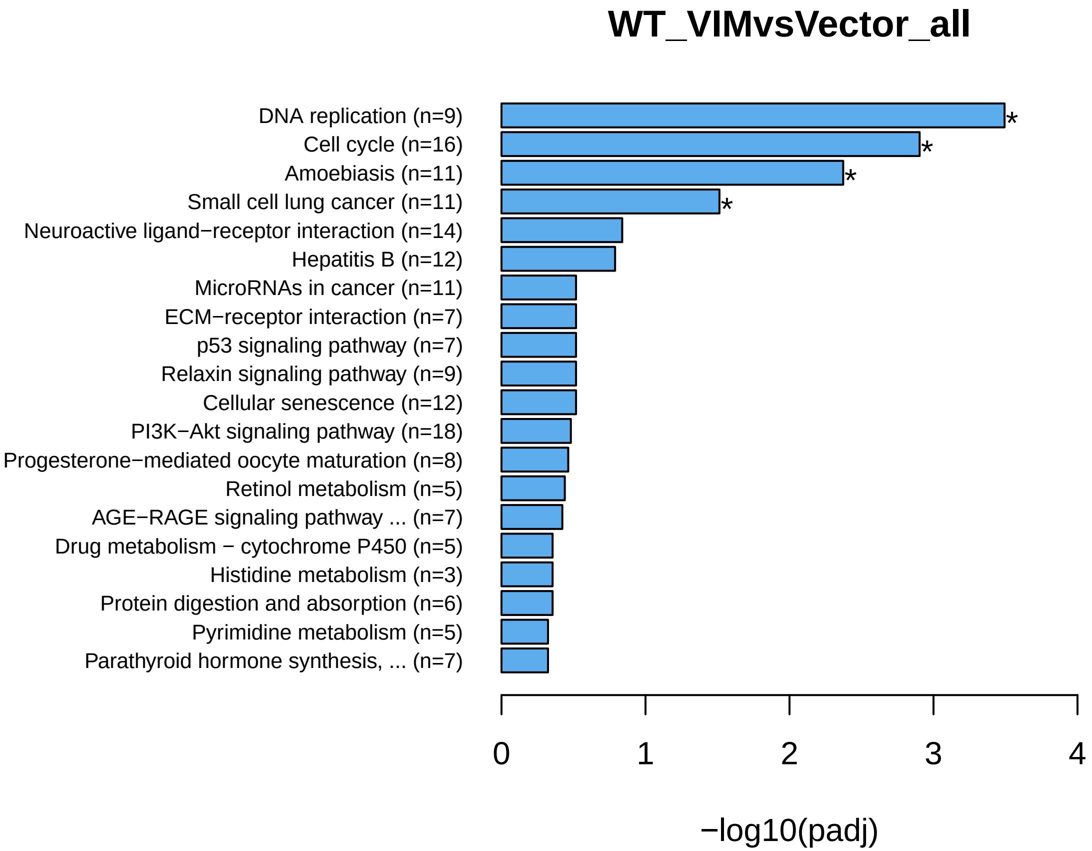
WT_VIMvsVector_all
DNA replication (n=9)
Cell cycle (n=16)
Amoebiasis (n=11)
Small cell lung cancer (n=11)
Neuroactive ligand−receptor interaction (n=14)
Hepatitis B (n=12)
MicroRNAs in cancer (n=11)
ECM−receptor interaction (n=7)
p53 signaling pathway (n=7)
Relaxin signaling pathway (n=9)
Cellular senescence (n=12)
PI3K−Akt signaling pathway (n=18)
Progesterone−mediated oocyte maturation (n=8)
Retinol metabolism (n=5)
AGE−RAGE signaling pathway ... (n=7)
Drug metabolism − cytochrome P450 (n=5)
Histidine metabolism (n=3)
Protein digestion and absorption (n=6)
Pyrimidine metabolism (n=5)
Parathyroid hormone synthesis, ... (n=7)
*
*
*
*
0
1
2
3
4
−log10(padj)
